# ﻿Italian natural history museums need specimen digitization and much more: a reply to Benvenuti et al.

**DOI:** 10.3897/zookeys.1137.97631

**Published:** 2022-12-22

**Authors:** Franco Andreone, Ferdinando Boero, Marco A. Bologna, Giuseppe M. Carpaneto, Riccardo Castiglia, Spartaco Gippoliti, Bruno Massa, Alessandro Minelli

**Affiliations:** 1 Museo Regionale di Scienze Naturali, Via G. Giolitti, 36, I-10123 Torino, Italy Museo Regionale di Scienze Naturali Torino Italy; 2 CNR-IAS, Stazione Zoologica Anton Dohrn, Villa Comunale, I-80121 Napoli, Italy CNR-IAS, Stazione Zoologica Anton Dohrn Napoli Italy; 3 Department of Sciences, Università Roma Tre, Viale G. Marconi, 446, I-00146 Roma, Italy Università Roma Tre Rome Italy; 4 Dipartimento di Biologia e Biotecnologie “Charles Darwin”, Università “La Sapienza” di Roma, Via A. Borelli, 50, I-00161 Roma, Italy Università “La Sapienza” di Roma Rome Italy; 5 Società Italiana per la Storia della Fauna “Giuseppe Altobello”, Viale Liegi, 48A, I-00198 Roma, Italy Società Italiana per la Storia della Fauna “Giuseppe Altobello” Rome Italy; 6 Dipartimento di Scienze agrarie, alimentari e forestali, Università di Palermo, Viale Scienze, 13, I-90128 Palermo, Italy Università di Palermo Palermo Italy; 7 Dipartimento di Biologia, Università di Padova, Via Ugo Bassi, 58B, I 35131 Padova, Italy Università di Padova Padova Italy

**Keywords:** Digitization, repository, scientific collections, specimens

## Abstract

We reply to the comments made by Benvenuti et al. (2022) about our paper on the Italian natural history museums and scientific collections and the need of a centralized hub and repository. While agreeing that digitization is a useful tool to valorize each museum and collection, we still believe that the suggestion of a centralized hub is valid and necessary. This would largely help in boosting coordination among museums, sharing personnel and resources, and in providing a place to deposit scientific collections that do not fit the scope of smaller museums.

We have read with great interest the letter of our colleagues in Florence Natural History Museum and National Research Council (CNR) (Benvenuti et al. 2022) about our proposal (Andreone et al. 2022) to create a centralized repository within the actions of the National Biodiversity Future Center (NBFC). In responding to the comments by these authors on our paper, we take the opportunity to provide more details about natural history museums (NHMs) in Italy and their effectiveness as research and conservation centers. NHMs are universally recognized as strongholds for taxonomic and environmental monitoring studies that are key tools for biodiversity inventories and species description and keeping trace of loss of taxa, climate change, and pathogenic information on zoonotic diseases and crop pests (Suarez and Tsutsui 2004; DuBay and Fuldner 2017). In their letter, the authors stressed the utility of DISSCo (Distributed System of Scientific Collections), a collaborative network which deals with the digitization of scientific collections and artifacts stored in museums to facilitate access of the international community to scientific collections, but disagreed with our suggestion of creating a new structure and/or upgrading an existing museum to act as a national repository/center. A more detailed explanation of the reasons that led these authors to dissent with such a proposal would have been helpful to better understand their criticism. We agree that digitization is needed, but we still believe that a centralized hub and repository is necessary.

Andreone et al. (2022) suggested that the past geo-political fragmentation of pre-unitarian Italy in small states led to the birth and persistence of small to medium sized scientific museums that were not capable of coalescing into a single larger museum or into an operational coordinated distributed museum of national importance (Andreone et al. 2014). Other countries (i.e., France, Hungary, UK, etc.) adopted the model of a large centralized museum where major scientific collections are deposited. In Germany, where the fragmentation in “Länder” (federate states) somehow mirrors the historical conditions of Italy, many museums aggregated within a network (i.e., Senckenberg Gesellschaft für Naturforschung) or became autonomous research institutions (typical in this sense is the name of the former Zoologisches Forschungsmuseum Alexander Koenig in Bonn, i.e., “Zoological Research Museum…”). Unfortunately, no step in this direction occurred in Italy, where museums are still mostly managed by local public administrations or universities.

While we are convinced that local museums are extremely helpful to raise awareness of biodiversity, we believe that they cannot serve (or only partly serve) to monitor and check biodiversity at a national level, a necessity that is crucial to safeguard animal and plant populations, species and ecosystems. Already in 1898 William Henry Flower stated that “it is only in national museums that the fulfillment of both functions [research and education] in fairly equal proportions can be expected. In almost all other museums the diffusion of knowledge, or popular education, will be the primary function” (Flower 1898: 38). For this reason, also we reaffirm that a centralized hub and repository is necessary, since it would support not only digitization, but also a wider array of activities that serve the mission of biodiversity research and conservation in Italy, namely the maintenance and increase of natural history collections and their continuative taxonomic revisions. To this aim, the training of technical, curatorial and taxonomic staff to be employed in a national museum is a priority to ensure long-term life to biodiversity research in Italy.

The letter also raised attention to the digitization of collections, stressing the importance of the DISSCo program, as already emphasized by Bartolozzi (2013) and Andreone et al. (2014). We totally agree with this claim, since digitization is an obvious tool to make collections more accessible and usable (Baird 2010). Finally, we also agree with the necessity of networking museum data, beginning with textual data, but also iconographic (i.e., with the use of 3D photographs or CT scans), and favoring the creation of an online catalogue of the primary types preserved in Italy. Eventually, large-scale digitization will become mandatory the path for most museums (Blagoderov et al. 2012), contributing to speed taxonomic research worldwide (Engel et al., 2021). This advantage became particularly tangible during the COVID19 pandemics, when it was difficult to visit collections, and online services were particularly precious. Such a digitization project is given high priority among the activities of the NBFC related to a national museum of biodiversity.

Unfortunately, the small size and generalized lack of personnel and fund shortfalls that affect most Italian NHMs hinders the capacity to advance the digitization of natural history collections and old catalogues. In fact, the number of curatorial personnel working in Italian NHMs is rarely over ten. In many museums, curators are too often considered technicians or polyvalent figures dedicated not only to collection management, but also (and sometimes eminently) to other activities such as exhibit preparation, communication, and administration. To make a comparison, the Natural History Museum in London has more than 80 curators, in addition to technicians, post-docs and other scientific personnel. Furthermore, due to personnel inadequacy, in Italy most museum collections are rarely taxonomically revised, new material is acquired through scientific expeditions only occasionally, and old catalogues are very rarely digitized and updated. Unfortunately, as stressed by several authors (i.e., Boero 2001; Vomero 2014), curators of Italian museums are too infrequently taxonomists and quite often devoted to other disciplines (i.e., ecologists, science historians, faunistics). Further, it should also be taken into account that Italian collections are rarely used as key taxonomic resources in international projects, and Italian museums were absent from the SYNTHESYS+ program (Bartolozzi 2013). Incidentally, the authors of the letter quote, beside Florence NHM, a few other entities adhering to DISSCo, such as the National Research Council (CNR), National Association of Scientific Museums (ANMS), National Academy of Sciences, National Academy of Entomology, Italian Society of Biogeography, Italian Paleontological Society, Italian Geological Society, and Italian Botanical Society (https://www.dissco.eu/it). With the exception of the CNR (which is also the coordinator of the constituting NBFC), the others are all scientific societies, with no primary functions in biodiversity collection and cataloguing. Of the more than 160 NHMs adhering to the CollMap initiative - a national census of major collections spread over Italian museums (Vomero 2013; http://www.anms.it/pagine/istituzioni) - only the Florence NHM is within the DISSCo network, whereas other large museums (like the ones in Genoa, Milan, Pisa, Turin, Rome, and Verona) are not included.

So far, DISSCo and other systems and networks, such as Global Biodiversity Information Facility (GBIF), VertNet, and Integrated Digitized Biocollections (iDigBio), are powerful tools which foster transnational collaboration and represent operative tools for the future, above all for the online study of specimens whose loans to scientists are increasingly difficult: the development of digital technologies will make it possible to send images online, without moving precious specimens. This is one of the necessary steps to get a better functionality and interconnection among Italian museums, but it should go together with a national coordination hub, so as to hire a critical mass of taxonomists and technicians. The need of a centralized repository for collections that do not fit the scopes of smaller museums, and/or do not have a primary exhibition value is also urgent. All this would also help, as Minelli (2013) already stressed, to share resources and better address research activities at a national and international level.
